# Does photobiomodulation therapy combined to static magnetic field (PBMT-sMF) promote ergogenic effects even when the exercised muscle group is not irradiated? A randomized, triple-blind, placebo-controlled trial

**DOI:** 10.1186/s13102-020-00197-6

**Published:** 2020-08-26

**Authors:** Caroline dos Santos Monteiro Machado, Heliodora Leão Casalechi, Adriane Aver Vanin, Jônatas Bezerra de Azevedo, Paulo de Tarso Camillo de Carvalho, Ernesto Cesar Pinto Leal-Junior

**Affiliations:** 1 Laboratory of Phototherapy and Innovative Technologies in Health (LaPIT), Nove de Julho University, Rua Vergueiro, 235/249, São Paulo, SP 01504-001 Brazil; 2 Postgraduate Program in Rehabilitation Sciences, Nove de Julho University, São Paulo, SP Brazil; 3 Physiotherapy Research Group, Department of Global Public Health and Primary Care, University of Bergen, Bergen, Norway

**Keywords:** Photobiomodulation therapy, Static magnetic field, Exercise performance, Muscle performance, Muscle recovery, Physiotherapy, Rehabilitation

## Abstract

**Background:**

The direct application of photobiomodulation therapy (PBMT) using low-level laser therapy (LLLT) and light emitting diodes (LEDs) combined with a static magnetic field (sMF) (PBMT-sMF) to target tissues is shown to improve muscle performance and recovery. Studies have reported possible PBMT effects when a local distant to the target tissue is irradiated. Notably, the extent of these effects on musculoskeletal performance and the optimal site of irradiation remain unclear, although this information is clinically important since these aspects could directly affect the magnitude of the effect. Therefore, we investigated the effects of local and non-local PBMT-sMF irradiations on musculoskeletal performance and post-exercise recovery before an eccentric exercise protocol.

**Methods:**

This randomized, triple-blind (participants, therapists and assessors), placebo-controlled trial included 30 healthy male volunteers randomly assigned to the placebo, local, and non-local groups. Active or placebo PBMT-sMF was applied to 6 sites of the quadriceps muscle of both legs. An eccentric exercise protocol was used to induce fatigue. The primary outcome was peak torque assessed by maximal voluntary contraction (MVC). The secondary outcomes were delayed onset muscle soreness (DOMS) measured by visual analogue scale (VAS), muscle injury assessed by serum creatine kinase activity (CK), and blood lactate levels. Evaluations were performed before the eccentric exercise protocol (baseline), as well as immediately after and 1, 24, 48, and 72 h upon protocol completion.

**Results:**

Ten volunteers were randomized per group and analysed for all outcomes. Compared to the placebo and non-local groups, irradiation with PBMT-SMF led to statistically significant improvement (*p* < 0.05) with regard to all variables in the local group. The outcomes observed in the non-local group were similar to those in the placebo group with regard to all variables.

The volunteers did not report any adverse effects.

**Conclusion:**

Our results support the current evidence that local irradiation of all exercised muscles promotes ergogenic effects. PBMT-sMF improved performance and reduced muscle fatigue only when applied locally to muscles involved in physical activity.

**Trial registration:**

NCT03695458. Registered October 04th 2018.

## Background

Photobiomodulation therapy (PBMT) refers to the application of electromagnetic radiation to biological tissues using low-power lasers or light-emitting diodes [1], which induces photochemical reactions in cells leading to a biomodulatory therapeutic effects [2, 3], without leading to ablative or thermal adverse reactions [4]. In recent years, this therapy has shown positive effects in the management of several musculoskeletal disorders and inflammatory conditions to promote pain relief and wound healing [5–11]. Many studies have reported that PBMT increases muscle performance, reduces fatigue, and improves muscle recovery in athletes, physically active, and sedentary individuals [12–22]. The main mechanism of action of PBMT include the interaction of photons with cytochrome c-oxidase, a mitochondrial photoreceptor [2], leading to greater transfer of electrons and consequently mitochondrial respiratory chain activation, which increases mitochondrial adenosine triphosphate (ATP) production [23]. Another mechanism of action for PBMT is attributed to increased microcirculation [24] and increased oxygen availability [25, 26].

Static magnetic fields (sMF) are also known to affect biological processes in the body. The sMF acts through the movement of electrically charged particles/electromagnetic waves on other body parts [27, 28]. Recent studies reported that sMF increases ATP production [27], and reduces oxidative stress [28]. Moreover, it was previously demonstrated that the combination of PBMT and sMF generates greater effects in cellular metabolism than the use of PBMT alone, through synergistic acceleration of cellular electron transfer [29].

In clinical scenario, many studies have also shown that PBMT-sMF has positive effects on muscle performance and post-exercise recovery in athletes and non-athletes [30–36]. Additionally, such intervention is known to improve the fatigue in the lower limbs of patients with chronic obstructive pulmonary disease [37, 38] and stroke [39]. Moreover, this therapy used in conjunction with different training programs has shown improved strength [33] and aerobic endurance [34], besides decreased deconditioning [40].

Although these effects have been observed following the local application of both PBMT and PBMT-sMF directly to the exercised muscles, it has been suggested that treatments at sites distant from the target tissue may also produce positive effects. On the other hand, Batista et al. [41] did not observe positive effects when PBMT was applied at a site distant from the target tissue.

Therefore, there is lack of consensus regarding the non-local effects of PBMT and PBMT-sMF. To date, we were able to identify only one clinical study that have investigated the non-local effects of PBMT on performance and recovery [42], and to the best of our knowledge there is none study that have investigated the non-local effects of PBMT-sMF on musculoskeletal performance and recovery, particularly with the use of biochemical markers to assess exercise-induced muscle injury. It would be important to investigate whether these effects also occur with non-local irradiation since it could have a direct impact on the magnitude of the effect, and as consequence, in clinical practice.

Therefore, this study has as null hypothesis that non-local treatment using PBMT-sMF is not able to promote between group differences in functional and biochemical markers related to skeletal muscle performance and post-exercise recovery, and that only local PBMT-sMF irradiation is able to promote such differences (alternative hypothesis). Thus, our aim was to investigate the effects of local and non-local PBMT-sMF irradiations on markers of musculoskeletal performance and post-exercise recovery before an eccentric exercise protocol to induce muscle fatigue.

## Methods

### Study design

A randomized, triple-blind (participants, therapists and assessors), placebo-controlled clinical trial approved by the research ethics committee (protocol number 2100849) was carried out at the Laboratory of Phototherapy and Innovative Technologies in Health (LaPIT), at Nove de Julho University (UNINOVE). It was prospectively registered at clinicaltrials.gov (NCT03695458). There were no changes to the original registration while conducting the study. All participants signed the informed consent before enrollment in the study.

### Sample characterization

Thirty sedentary male participants between 18 and 35 years old were recruited. All participants who agreed to participate in the study signed an informed consent form. The calculation of sample size (with beta value of 20% and alpha of 5%) was based on the study of Antonialli et al. [30] which used the same PBMT-sMF device and observed increased maximal voluntary contraction (MVC; our primary outcome) at 96 h post-exercise (336.88 ± 27.92 Nm) compared to baseline (286.63 ± 38.86 Nm). Thus, the calculation resulted in 10 volunteers per group (30 volunteers in total).

**Fig. 1:**
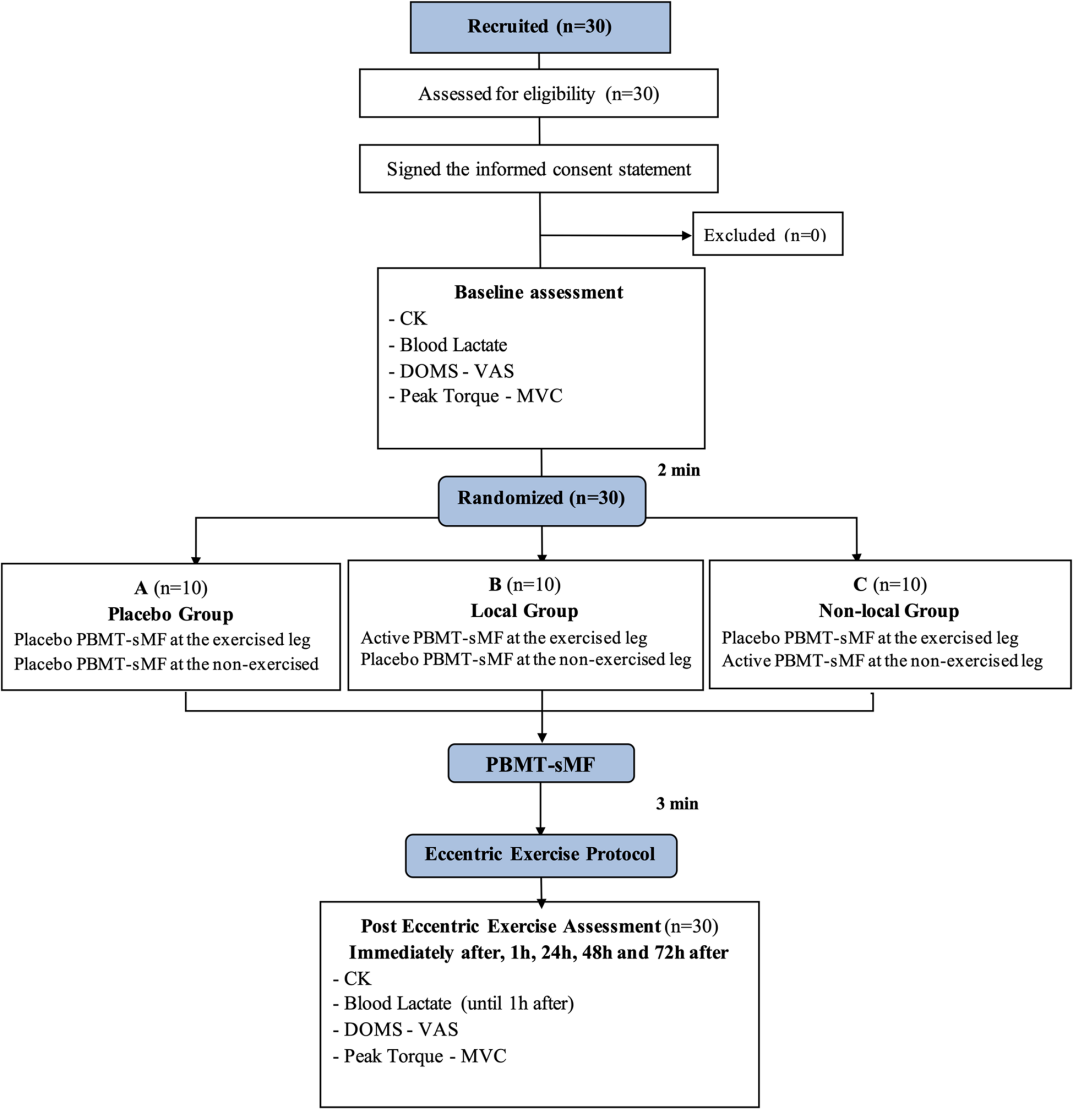
CONSORT flowchart

Even though there were no dropouts in the study, the intention-to-treat analysis protocol, established a priori, was followed. The flowchart CONSORT (Consolidated Standards of Reporting Trials) shows the procedures of this study (Fig. 1).

### Inclusion and exclusion criteria

The study included male participants, aged between 18 and 35 years, with different skin pigmentations and who performed up to 1 exercise session weekly. With no pre-existing musculoskeletal injuries in the hips or knees in the 2 months prior to the study.

Individuals who did not meet the above criteria were excluded from the study, such as those using food supplements, those who had chronic joint disease in the non-dominant lower limb, or who had musculoskeletal injury during the study.

### Experimental groups and randomization

The 30 volunteers were randomly assigned to 3 experimental groups (*n* = 10 per group) according to the type of therapy and limb to be irradiated as described below.

- Placebo group: irradiation with placebo PBMT-sMF, bilaterally to the anterior thigh muscles of both lower limbs;
- Local group: irradiation with active PBMT-sMF to the anterior thigh muscles of the exercised lower limb and irradiation with placebo PBMT-sMF to the anterior thigh muscles of the non-exercised limb;
- Non-local group: irradiation with placebo PBMT-sMF to the anterior thigh muscles of the exercised lower limb and irradiation with active PBMT-sMF to the anterior thigh muscles of the non-exercised limb;

Randomization was performed 2 min after baseline evaluation by a researcher who was not involved in treatments of assessments of participants. Randomization labels were created through the random.org website, and a series of sealed, opaque, and numbered envelopes were used to ensure confidentiality and to determine to which experimental group each volunteer was to be allocated.

The PBMT-sMF device was programmed by a researcher who did not participate in any of the stages of data collection and analysis. He was instructed not to disclose the programing until the study was completed. The device contained distinct programs, corresponding to active PBMT-sMF or placebo. The different programs looked the same regarding light, sound and application time, providing adequate researcher and volunteer blindness. In addition, the volunteers wore opaque glasses which assisted in the blinding. The professionals who evaluated MVC, CK, VAS, lactate and the one who applied the eccentric protocol was not the same one who performed the active and placebo PBMT-sMF treatments. All these procedures were performed to ensuring triple-blind design.

### Procedures

#### Creatine kinase (CK) activity assessment

Blood samples were collected (5 ml by anterior cubital vein puncture) prior to stretching and warm-up (baseline) immediately after, 1, 24, 48 and 72 h after the eccentric exercise protocol. Fifteen minutes after collection the samples were centrifuged at 3000 rpm for 20 min and the supernatant serum was stored and kept at − 80 °C until analysis.

Creatine kinase (CK) enzymatic activity as an indirect marker of muscle damage was analyzed by spectrophotometry using specific reagent kits (Labtest® - Brazil) following the manufacturer’s instructions with the collected blood samples.

#### Blood lactate assessment

Blood samples were collected from the volunteers’ fingertips prior to stretching and warm-up (baseline), immediately after, and 1 h after the eccentric exercise protocol. After asepsis a puncture was performed with a disposable lancet, the first drop of blood was discarded to prevent contamination, then 25 μl were collected for biochemical analysis by the electroenzymatic method according to the instructions of the portable lactate analyzer manufacturer (Accutrend Lactate Plus Roche, Roche Diagnostics GmbH, Mannheim, Germany). The analyzer has a variation coefficient between 1.8 and 3.3% (intraclass correlation [ICC] *r* = 0.999), with good reliability for intra/inter-analyzers and between test strips [43].

#### Evaluation of delayed onset muscle soreness (DOMS)

DOMS of exercised lower limb was evaluated by the Visual Analogue Scale (VAS), which consists of a 100 mm line. At the beginning of the line, number 0 corresponds to no pain and at the end, 100 corresponds to the worst possible pain. The volunteers were instructed to draw a line where their pain best fit at the moment. We decided to use this method since previous research showed high test-retest reliability for VAS in assessment of DOMS [44]. Assessments were performed prior to stretching and warm- up (baseline), immediately after, 1, 24, 48 and 72 h after the eccentric exercise protocol was performed.

#### Stretching and warm-up

Before starting the isokinetic protocol, the volunteers performed 3 sets of 60 s of active stretching of the knee extensors/hip flexors muscles, bilaterally. Next, they walked for 5 min at 6 km/h on a treadmill as a general warm-up activity.

#### Maximum voluntary contraction (MVC) test

The muscle strength assessment and execution of the eccentric exercise protocol for fatigue induction was performed using an isokinetic dynamometer (System 4 model, Biodex Medical Systems®, Inc., Shirley, NY, USA). Currently considered as gold standard method for assessing the maximum capacity for muscle strength generation and musculoskeletal performance [45, 46].

Immediately after stretching and warm-up exercises, the volunteers performed the maximum voluntary contraction test (MVC). They were positioned on the seat of the isokinetic dynamometer with a 100° angle between the trunk and the hip, and fixed to the dynamometer seat by belts. The non-dominant leg was positioned at 60° of knee flexion (0° corresponding to total knee extension) with the dynamometer axis parallel to the center of the knee joint.

The MVC test consisted of three isometric contractions of knee extensors of the non-dominant leg lasting 5 s with 30-s intervals between contractions. The highest peak torque value obtained from the three contractions was used for statistical analysis. The volunteers were instructed on how to perform the exercise prior to the beginning of the MVC protocol and during the test they were verbally encouraged by the same researcher in all assessments. The MVC test was performed previously (baseline), immediately after, 1, 24, 48 and 72 h after the eccentric exercise fatigue protocol.

#### Photobiomodulation therapy and static magnetic field (PBMT-sMF)

**Fig. 2:**
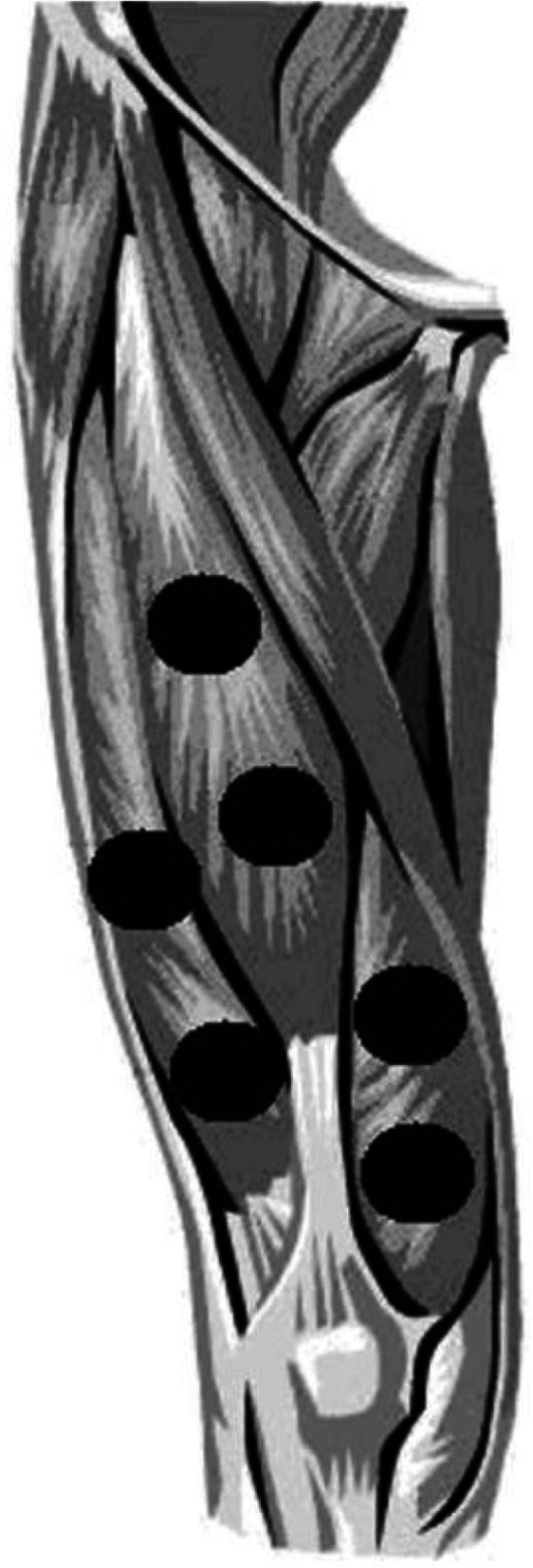
Treatment sites at knee extensor muscles

Active PBMT-sMF or active placebo was applied bilaterally, 2 min after baseline assessment (pre-exercise MVC test). The application technique used was direct skin contact and slight pressure, in 6 sites covering the knee extensor muscles (quadriceps): 2 lateral, 2 medial and 2 central (Fig. 2).

PBMT and sMF were applied simultaneously since these two modalities were part of the same therapeutic device. For such, cluster probes containing 12 diodes and a static magnetic field were used. The cluster probes consisted in 4 laser diodes of 905 nm (0.3125 mW average power, 12.5 W peak power for each diode), 4 LEDs of 875 nm (17.5 mW average power for each diode), 4 LEDs of 640 nm (15 mW average power for each diode) and a static magnetic field of 35 mT (device manufactured by Multi Radiance Medical®, Solon - OH, USA), the total dose was 180 J per thigh (for the active PBMT-sMF). Given the extensive area of irradiation employed in the present work, the use of cluster probes was paramount to optimize the therapy application. The choice of treatment parameters and locations for PBMT-sMF were based on previous studies using the same equipment [30, 33]. The complete description of the parameters is given in the Table 1.

**Table 1:** Parameters for PBMT-sMF

Number of lasers	4 Super-pulsed (infrared)
Wavelength (nm)	905 (±1)
Frequency (Hz)	250
Peak power (W) - each	12.5
Average mean optical output (mW) - each	0.3125
Power density (mW/cm^2^) - each	0.71
Energy density (J/cm^2^) - each	0.162
Dose (J) - each	0.07125
Spot size of laser (cm^2^) - each	0.44
Number of red LEDs	4 Red
Wavelength of red LEDs (nm)	640 (±10)
Frequency (Hz)	2
Average optical output (mW) - each	15
Power density (mW/cm^2^) - each	16.66
Energy density (J/cm^2^) - each	3.8
Dose (J) - each	3.42
Spot size of red LED (cm^2^) - each	0.9
Number of infrared LEDs	4 Infrared
Wavelength of infrared LEDs (nm)	875 (±10)
Frequency (Hz)	16
Average optical output (mW) - each	17.5
Power density (mW/cm^2^) - each	19.44
Energy density (J/cm^2^) - each	4.43
Dose (J) - each	3.99
Spot Size of LED (cm^2^) - each	0.9
Static Magnetic Field (sMT)	35
Irradiation time per site (sec)	228
Total dose per site (J)	30
Total dose applied in muscular group (J)	180
Aperture of device (cm^2^)	20
Application mode	Cluster probe held stationary in skin contact with a 90-degree angle and slight pressure

#### Eccentric exercise protocol

Three minutes after the end of active or placebo PBMT-sMF treatment the volunteers performed the eccentric contractions protocol to induce fatigue. It consisted of 75 eccentric isokinetic contractions of the non-dominant lower limb knee extensor muscles (5 sets of 15 repetitions with 30 s interval between each set), with a velocity of 60° sec^1^ (both in the eccentric and concentric phase of the movement) and 60° range of motion (between 30° and 90° of knee flexion). At each contraction, the dynamometer automatically positioned the knees (passively) at 30° and then flexes it to 90°. The efficiency of this protocol has previously been demonstrated to induce muscle damage induced by exercise [16, 30, 32].

The volunteers were instructed to resist the knee flexion movement imposed by the dynamometer with maximum force and during the protocol, and they were verbally encouraged by a single assessor blinded to patients’ allocation to the different experimental groups.

### Statistical analysis

The primary outcome of this study was MVC, and the secondary outcomes were CK activity, blood lactate, and DOMS. The intention-to-treat analysis was followed a priori, and all data were analysed by a blinded researcher who was not involved in the data collection. The findings were tested for normality using the Kolmogorov-Smirnov test and were determined to have a normal distribution. Data were expressed as the mean and standard deviation, and a mixed design ANOVA (repeated design for time, non-repeated design for group) was performed to test between-group differences at each timepoint, followed by the Bonferroni post hoc test. Data were analyzed in terms of the absolute values and the percentage of change based on the values established at baseline. The analysis of the percentage of change was performed to both decrease the data variability, and to provide a better representation of the magnitude of changes observed from the absolute data. The significance level was set at *p* < 0.05. In the graphs, data are expressed as the mean and standard error of the mean (SEM).

## Results

The recruitment of volunteers was performed between January 2019 and May 2019. Thirty male volunteers with mean age 26.83 years (± 6.02), height 175.67 cm (± 7.95) and body mass 73.03 kg (± 12.59) completed all procedures of the study, there were no dropouts. Ten volunteers were randomized per group and analyzed for all outcomes by original assigned groups. All patients received treatment according to the randomized allocation. The volunteers did not report any adverse effects. Data were analyzed and no statistically significant differences (*p* > 0.05) at baseline were observed between all experimental groups according for MVC, CK, blood lactate and DOMS variables. However, statistically significant differences (*p* < 0.05) were observed after the baseline between the placebo group and the local group for MVC, CK activity, blood lactate and DOMS. The full description of these data in absolute values, expressed as mean, standard deviation and confidence intervals are shown in Table 2, as well as the differences among groups.

**Table 2:** Functional and biochemical markers of performance and recovery in absolute values. Values are expressed as mean, standard deviations (± SDs) and confidence intervals

Variable	Group	Baseline	Immediately after	1 h	24 h	48 h	72 h
**MVC** (Nm)	Placebo	228.28 (± 30.27)	177.21 (± 27.33)	172.98 (± 29.38)	171.21 (± 29.40)	167.65 (± 29.80)	193.15 (± 29.95)
		[209.52–247.04]	[160.27–194.14]	[154.77–191.19]	[152.99–189.43]	[149.18–186.12]	[174.59–211.71]
	Local	231.72 (± 34.74)	213.54 (± 32.58)^**b**^	203.03 (± 30.23)^**b**^	213,74 (± 32.48)^**a,b**^	225.30 (± 29.45)^**a,b**^	249.59 (± 37.58)^**a,b**^
		[210.19–253.25]	[193.35–233.73]	[184.29–221.77]	[193.61–233.87]	[207.05–243.55]	[226.30–272.88]
	Non-local	213.11 (± 30.04)	171.93 (± 29.88)	155.63 (± 25.18)	164.56 (± 21.51)	181.08 (± 30.32)	178.74 (± 21.74)
		[194.49–231.73]	[153.41–190.45]	[140.02–171.24]	[151.23–177.89]	[162.29–199.87]	[165.27–192.21]
**CK** (U.L^−1^)	Placebo	43.87 (± 16.28)	43.31 (± 15.16)	58.51 (± 20.73)	162.31 (± 56.01)	234.97 (± 87.49)	233.60 (± 94.19)
		[33.78–53.96]	[33.91–52.71]	[45.66–71.36]	[127.60–197.02]	[180.74–289.20]	[175.22–291.98]
	Local	38.20 (± 11.29)	34.70 (± 12.83)	35.38 (± 15.34)	69.83 (± 30.35) ^**a,b**^	74.80 (± 35.68)^**a,b**^	54.53 (± 28.85) ^**a,b**^
		[31.20–45.20]	[26.75–42.65]	[25.87–44.89]	[51.02–88.64]	[52.69–96.91]	[36.65–72.41]
	Non-local	50.49 (± 12.89)	49.74 (± 15.09)	59.62 (± 16.02)	168.98 (± 61.69)	252.83 (± 118.01)	296.81 (± 88.28)
		[42.50–58.48]	[40.39–59.09]	[49.69–69.55]	[130.74–207.22]	[179.69–325.97]	[242.09–351.53]
**DOMS** (VAS - mm)	Placebo	0 (± 0)	13.10 (± 7.56)	37.40 (± 17.42)	49.70 (± 14.25)	52.30 (± 20.12)	50.60 (± 20.38)
		[0–0]	[8.40–17.80]	[26.60–48.20]	[40.80–58.60]	[39.80–64.80]	[38.00–63.20]
	Local	0 (± 0)	21.40 (± 14.17)	12.30 (± 14.28) ^**a,b**^	16.60 (± 15.59) ^**a,b**^	23.20 (± 17.83) ^**a,b**^	20.40 (± 15.49) ^**a,b**^
		[0–0]	[12.60–30.30]	[3.40–21.20]	[6.90–26.30]	[12.20–34.20]	[10.80–30.00]
	Non-local	0 (± 0)	21.70 (± 11.45)	42.40 (± 25.65)	54.50 (± 30.70)	65.90 (± 27.98)	57.20 (± 31.14)
		[0–0]	[14.60–28.80]	[26.50–58.30]	[35.50–73.50]	[48.50–83.30]	[37.90–76.50]
**Lactate** (mmol. L^− 1^)	Placebo	2.45 (± 0.83)	4.73 (± 1.47)	2.30 (± 0.84)			
		[1.94–2.96]	[3.82–5.64]	[1.78–2.82]	–	–	–
	Local	2.56 (± 0.44)	3.35 (± 0.76) ^**a,b**^	2.14 (± 0.33)			
		[2.29–2.83]	[2.88–3.82]	[1.94–2.34]	–	–	–
	Non-local	2.06 (± 0.52)	4.62 (± 1.67)	2.31 (± 0.60)			
		[1.74–2.38]	[3.58–5.66]	[1.94–2.68]	–	–	–

^**a**^Different compared to Placebo group (*p* < 0.05)

^**b**^Different compared to Non-local group (*p* < 0.05)

**Fig. 3:**
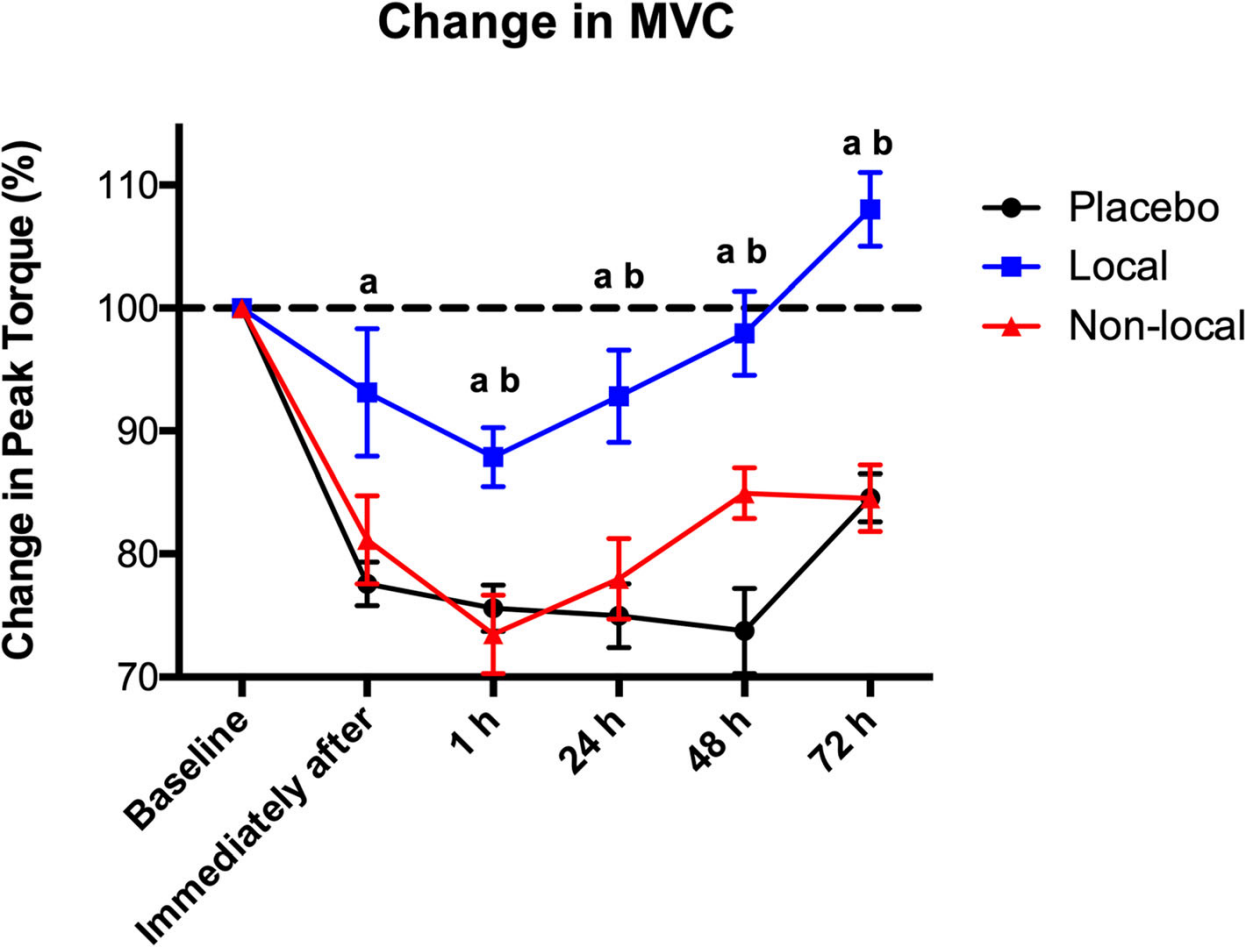
Percentage of change in MVC. ^a^ indicates statistical difference (*p* < 0.05) compared to placebo group; ^b^ indicates statistical difference (*p* < 0.05) compared to of non-local group

Regarding the percentage of change for MVC, a statistically significant improvement in the peak torque (*p* < 0.05) was observed immediately after the eccentric exercise protocol in the local group (93.13% ± 16.44) compared to the placebo group (77.56% ± 5.62), the statistically significant difference (*p* < 0.05) was also observed in all timepoints. A statistically significant difference (*p* < 0.05) was also observed at 1 h after the eccentric exercise protocol between the local group (87.87% ± 7.58) and the non-local group (73.46% ± 10.08). This difference remained at 24, 48 and 72 h after the eccentric exercise protocol, as shown in Fig. 3.

**Fig. 4:**
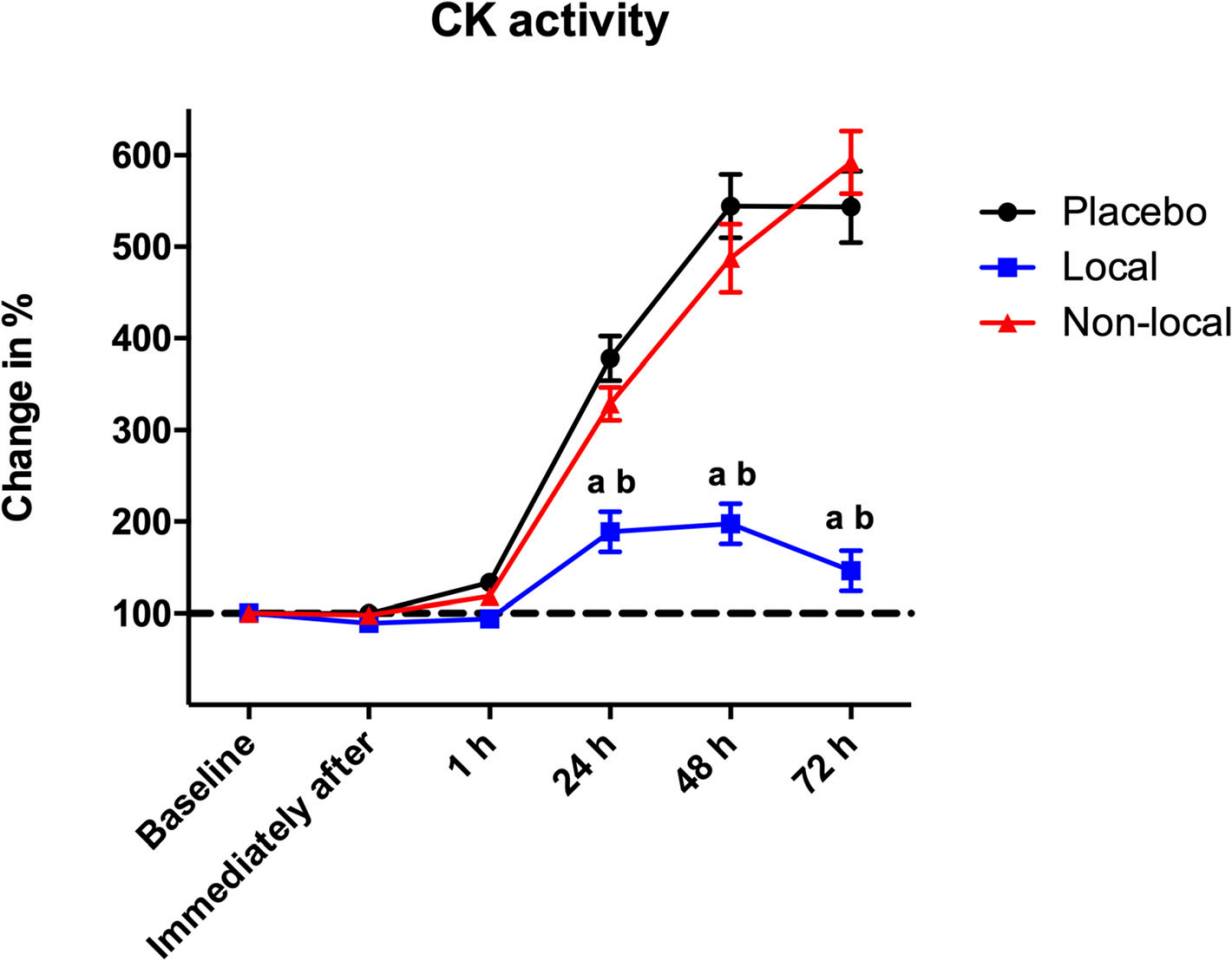
Percentage of change in CK activity. ^a^ indicates statistical difference (*p* < 0.05) compared to placebo group; ^b^ indicates statistical difference (*p* < 0.05) compared to of non-local group

Figure 4 shows a statistically significant lower increase (*p* < 0.05) in percentage of change for CK activity at 24 h after the eccentric exercise between the local group (188.91% ± 69.64) compared to the placebo (378.27% ± 76.42) and non-local groups (328.90% ± 57.02). This difference was also observed 48 h and 72 h after the eccentric exercise protocol.

**Fig. 5:**
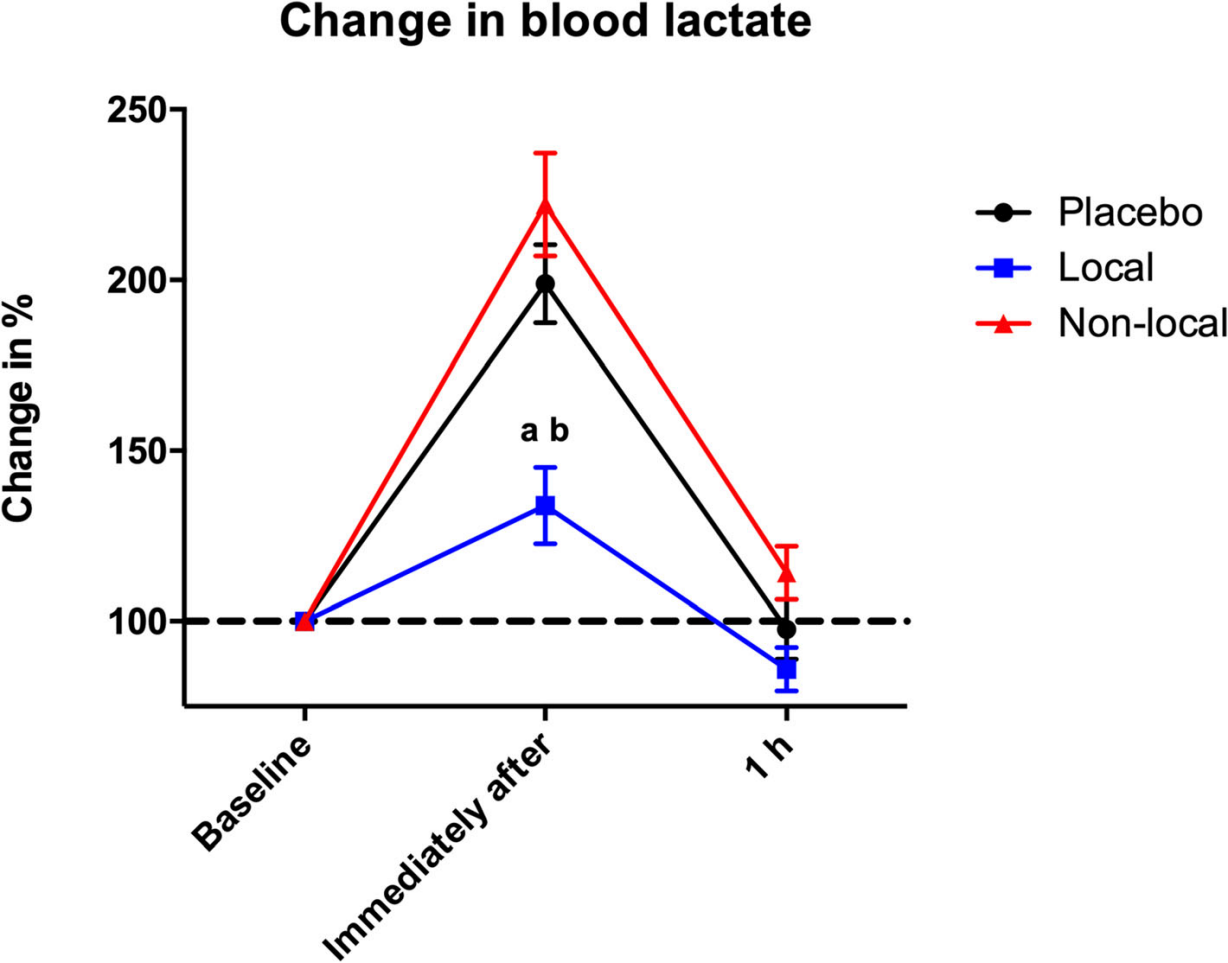
Percentage of change in blood lactate levels. ^a^ indicates statistical difference (*p* < 0.05) compared to placebo group; ^b^ indicates statistical difference (*p* < 0.05) compared to of non-local group

The change in blood lactate levels showed a statistically significant difference (*p* < 0.05) right after the eccentric exercise protocol for the local group (133.85% ± 35.59) compared to the placebo (198.98% ± 36.39) and non-local groups (222.21% ± 47.80). No statistically significant differences were observed among the groups at 1 h after the eccentric exercise protocol (Fig. 5).

## Discussion

This study showed that PBMT-sMF did not increase performance or reduce strenuous exercise-induced fatigue when applied to sites distant from the exercised muscle. This finding supports the hypothesis that PBMT-sMF should be applied locally to muscles that will be exercised and concurs with the results reported by several previous studies [30, 33–36], as well as the results of current systematic reviews and meta-analyses discussing this subject and the recently published guidelines [1, 14, 15]. To summarize, the maximal voluntary contraction (MVC) in the local group was similar to that reported by Antonialli et al. [30] who observed that application of local PBMT-sMF using an optimal dose (180 J/thigh) improves muscle performance. Moreover, we observed that compared to baseline levels, muscle strength in the local group recovered completely within 48 h after the eccentric exercise. Muscle performance in the local group at 72 h was higher than that recorded at baseline evaluation, indicating better performance and more effective muscle recovery.

The eccentric exercise protocol effectively induced muscle fatigue, as confirmed by the fact that the placebo group showed decreased performance (based on MVC assessment) after exercise and increased muscle damage and fatigue. These observations were verified by estimation of blood markers (creatine kinase - CK, and lactate) and muscle pain. The group that received local PBMT-sMF showed lesser muscle damage and a minimal increase in serum CK activity within the first 24 h after exercise. The local group also showed reduced blood lactate levels immediately after exercise. The estimation of blood lactate shows good clinical applicability and is widely used for performance analysis in sport settings and because the measurement of this biochemical marker is easy and cost effective [47, 48].

Compared to the placebo and the non-local groups, the local group showed a statistically significant reduction in exercise-induced pain [49, 50] at 1, 24, 48, and 72 h after completion of the eccentric exercise protocol. These results are similar to those reported by previous studies using PBMT-sMF [30, 32], suggesting that PBMT-sMF could increase local microcirculation [24] and effectively removes blood metabolites, helping to reduces fatigue, and accelerates muscle recovery after exercise [35]. Clinically, attenuating the fatigue perception process is important for muscle recovery because it enables individuals to rapidly return to physical activities with lesser motor impairment [51].

Our results with regard to muscle strength and recovery from fatigue concur with those reported by Ferreira Junior et al. [42] using PBMT only, which observed that compared with placebo irradiation, local PBMT irradiation to the exercised leg led to an 11.3% improvement in functional performance. Notably, our results showed that compared with placebo irradiation, local PBMT-sMF irradiation of the exercised leg led to 20.07% (immediately after eccentric protocol) to 32.78% (48 h after eccentric protocol) improvement in functional performance. In our view, the greater improvement observed in our study compared with that reported by Ferreira Junior et al. [42] could be attributed to the combination of PBMT and sMF instead the use of PBMT only, as previously reported [52]. Moreover, Ferreira Junior et al. [42] did not observe positive effects of PBMT as single therapy on blood lactate levels, which can be attributable to differences between the fatigue induction protocols implemented in these studies. Furthermore, Ferreira Junior et al. [42] do not assessed the CK activity, which is an important biochemical marker of exercise-induced muscle injury [53]. Therefore, to determine the magnitude of the effects of PBMT-sMF on skeletal muscles and ensure consistent evaluation and robust results, we assessed CK activity, since this biochemical marker is commonly used in clinical practice to assess muscle status [53].

Some studies have reported a possible effect of PBMT when applied distant from the target tissue [54], suggesting that this effect may optimize the length of treatment and as consequence, obviate the need to irradiate all muscle groups involved in the exercise for instance. This possible effect is attributed to the direct release of nitric oxide from hemoglobin and nitrosylated myoglobin [55] causing vasodilatation, increased blood flow, and faster recovery of muscles throughout the body. On the other hand, it is also known that PBMT causes biological changes at the cellular level secondary to interactions between photons and cytochrome c-oxidase, a mitochondrial enzyme, which is a cellular organelle that is not present in the blood. The duration of this interaction should be adequate to last until complete application of the ideal dose, thereby achieving the desired effect on the target tissue [2]. It must be emphasized that PBMT or PBMT-sMF can increase or decrease cellular activity, based on the therapeutic window and the applied dose [2, 29]. Moreover, according to the current evidence and guidelines at least 30 s are required to promote ergogenic effects on muscles, and the application should be performed in a stationary position [1, 15].

We observed that PBMT-sMF did not produce effects when applied to sites distant from the target area. This is an interesting finding that highlights an important aspect associated with the safety of this therapy and also the possibility of adverse effects, such as the non-local effects caused by certain drugs [56]. For example, some non-steroidal anti-inflammatory drugs (NSAIDs) inhibit cyclo-oxygenase-2 (COX-2) activity, thereby reducing inflammation and pain [56]. However, the NSAID-induced systemic reduction in COX-2 activity may cause serious adverse effects [56], such as changes in gastric mucosal protection [57, 58] or a high risk of myocardial infarction [59].

The lack of effects in areas distant from the irradiated site leads to the conclusion that the interactions between PBMT-sMF and tissues occur only at the irradiated sites. This observation confirms that in addition to its aforementioned benefits, PBMT-sMF is a safe therapeutic alternative because it avoids effects, and as consequence adverse effects, in tissues distant from the application site. Therefore, our results highlight the relevance of PBMT-sMF in clinical practice and reiterate the importance of establishing an optimal approach for its application using the appropriate parameters and irradiation technique. Moreover, our findings will guide therapists with the correct application technique to target the muscles involved in exercise activity to ensure improved performance and reduced fatigue [1]. In essence, partial irradiation or irradiation of muscles not involved in a specific activity seems to be ineffective.

A limitation of the present study is that the possible effects of PBMT-sMF were not evaluated in tissues other than muscles in areas distant from the irradiated site. Therefore, further studies are needed to determine whether PBMT-sMF affects tissues other than muscles at sites distant from the irradiated area.

## Conclusion

Our results show that PBMT-sMF irradiation can improve performance and reduce muscle fatigue only when applied locally to exercised muscles. No positive effects were observed when a local distant from the exercised muscles was irradiated, indicating that muscles involved in physical activity should undergo local irradiation to achieve ergogenic effects of PBMT-sMF.

## Data Availability

The datasets used and/or analyzed during the current study are available from the corresponding author on reasonable request.

## Abbreviations

ATP: Adenosine triphosphate
COX2: Cyclooxygenase 2
CK: Creatine kinase
DOMS: Delayed onset muscle soreness
LEDs: Light emitting diodes
LLLT: Low-level laser therapy
LPL: Low power laser
MVC: Maximum voluntary contraction
NSAIDs: Non-steroidal anti-inflammatory drugs
PBMT: Photobiomodulation therapy
sMF: Static magnetic field
VAS: Visual analogue scale
